# APOE‐ε4 associations with smartphone‐based cognitive markers

**DOI:** 10.1002/alz.090766

**Published:** 2025-01-09

**Authors:** Anna M Rosická, Sol Fittipaldi, Eoghan Gallagher, Anna K Hanlon, Agustín Ibáñez, Katie Bridgeman, Graciela Muniz‐Terrera, Karen Ritchie, Craig W Ritchie, John T O'Brien, Ivan Koychev, Brian Lawlor, Lorina Naci, Claire M Gillan

**Affiliations:** ^1^ Trinity College Dublin, Dublin Ireland; ^2^ Global Brain Health Institute, Trinity College Dublin, Dublin Ireland; ^3^ BrainLat Institute, Santiago, Santiago Chile; ^4^ Edinburgh Dementia Prevention, University of Edinburgh, Edinburgh United Kingdom; ^5^ Institut for Neurosciences of Montpellier, University Montpellier, National Institute for Health and Medical Research, Montpellier France; ^6^ Scottish Brain Sciences, Edinburgh United Kingdom; ^7^ Department of Psychiatry, University of Cambridge, Cambridge United Kingdom; ^8^ University of Oxford, Oxford, Oxon United Kingdom

## Abstract

**Background:**

Smartphone‐based assessments are a promising tool for early detection of cognitive decline in midlife. Previous research has shown such cognitive markers can be sensitive to a range of potentially modifiable dementia risk factors even in healthy adults. However, their sensitivity to genetic risk factors like APOE‐ε4 is likely to differ by cognitive domain, with evidence of strong negative effects on wayfinding tasks but mixed for other domains. We investigated the associations between a set of previously validated smartphone‐based cognitive markers and APOE‐ε4.

**Method:**

*N* = 87 cognitively unimpaired, APOE‐genotyped participants (aged 44‐67, 57.5% female) of the PREVENT study completed a self‐administered module in the smartphone application Neureka, including gamified cognitive tasks and memory self‐evaluation (completion rates: 81%–99%). Controlling for age, gender, education, and parental history of dementia, we compared APOE‐ε4 carriers (*n* = 35) with non‐carriers in their performance on five cognitive measures: model‐based planning, visual working memory, processing speed (∼Trails A), cognitive flexibility (∼Trails B), and subjective memory problems. We conducted receiver operating characteristic (ROC) analysis to assess if APOE‐ε4 status can be predicted from norm‐adjusted cognitive scores, i.e., the difference between observed cognitive performance and what we would predict for given demographics based on an additional benchmark sample (*N* = 3,376, aged 18‐84, 65% female).

**Result:**

In univariate analyses, APOE‐ε4 carriers did not significantly differ from non‐carriers in their demographic characteristics or cognitive scores except for visual working memory, which was better in carriers (*t*(63.226) = 2.245; *p* = .028). After controlling for covariates, APOE‐ε4 status was not significantly linked to, which was better in APOE‐ε4 carriers (*β*[bootstrapped 95% CI] = ‐0.29[‐0.53,‐0.06]; *t* = ‐2.30; *p* = .025). In ROC‐analysis, norm‐adjusted visual working memory scores modestly differentiated between carriers and non‐carriers (AUC(SE) = .648(0.067); bootstrapped 95% CIs = 0.515–0.774).

**Conclusion:**

The non‐significant associations between APOE‐ε4 and most cognitive markers suggest limited sensitivity to genetic risk. Interestingly, APOE‐ε4 carriers outperformed non‐carriers in visual working memory, which could be due to antagonistic pleiotropy effects, but needs to be replicated in larger samples. The current study shows limited evidence for the clinical utility of our cognitive markers as indicators of genetic risk in middle‐aged cognitively unimpaired adults.

